# Insights into Dysregulated Neurological Biomarkers in Cancer

**DOI:** 10.3390/cancers16152680

**Published:** 2024-07-27

**Authors:** Elisa Duranti, Chiara Villa

**Affiliations:** School of Medicine and Surgery, University of Milano-Bicocca, 20900 Monza, Italy; e.duranti@campus.unimib.it

**Keywords:** biomarkers, cancer, neurodegenerative diseases

## Abstract

**Simple Summary:**

The connection between neurodegenerative diseases (NDs) and cancer has sparked a growing interest in biomedical research. Cancer cells show alterations in proteins linked to ND (tau, amyloid-β, α-synuclein, SOD1, and TDP-43). This review offers an updated summary of the biological role of these proteins in cancer. Specifically, we explore the effects of these proteins on cancer biology and how they affect these processes. Finally, we address the challenges and opportunities of targeting these proteins in the development of new cancer treatments.

**Abstract:**

The link between neurodegenerative diseases (NDs) and cancer has generated greater interest in biomedical research, with decades of global studies investigating neurodegenerative biomarkers in cancer to better understand possible connections. Tau, amyloid-β, α-synuclein, SOD1, TDP-43, and other proteins associated with nervous system diseases have also been identified in various types of solid and malignant tumors, suggesting a potential overlap in pathological processes. In this review, we aim to provide an overview of current evidence on the role of these proteins in cancer, specifically examining their effects on cell proliferation, apoptosis, chemoresistance, and tumor progression. Additionally, we discuss the diagnostic and therapeutic implications of this interconnection, emphasizing the importance of further research to completely comprehend the clinical implications of these proteins in tumors. Finally, we explore the challenges and opportunities in targeting these proteins for the development of new targeted anticancer therapies, providing insight into how to integrate knowledge of NDs in oncology research.

## 1. Introduction

Neurodegenerative disorders (NDs) and cancer are often considered to be at opposite extremes in terms of disease mechanisms [1]. While NDs, such as Alzheimer’s disease (AD), Parkinson’s (PD), and amyotrophic lateral sclerosis (ALS), are associated with faster rates of spontaneous cell loss than aging, cancer is distinguished by enhanced resistance cell death [2,3]. However, these opposite directions suggest the possibility of shared etiopathogenic mechanisms because changes to these regulatory systems could promote either the death of cells or their proliferation [4,5]. Moreover, aging, defined as the decline of physiological processes essential for survival and reproduction, is believed to be one of the main risk factors for both NDs and cancer, thus highlighting a complex and intriguing interrelation between them [6]. This relationship is further confirmed by several epidemiological studies that show a low cancer rate in patients affected by NDs and vice versa, suggesting that susceptibility to one disease may protect against the other [7,8,9,10,11,12,13]. Similarly, cancer survivors are more susceptible to developing other age-related disorders, such as osteoarthritis, stroke, non-neurodegenerative dementia, and macular degeneration [10,14]. Nevertheless, this inverse association is not consistent across all tumor types, with PD patients having an increased incidence of malignant melanoma and breast and prostate cancers [15,16,17]. This intricate interplay can also be altered by cancer treatment: some research found that chemotherapy-treated individuals who have survived breast cancer may have less connectivity and organization in their white matter compared to healthy controls [18], while other studies link chemotherapy to a decreased risk of developing AD [14].

While the molecular and cellular mechanisms driving NDs and cancer differ, both conditions underscore the vulnerability of normal cellular processes. NDs target neurons and their complex networks, disrupting communication and leading to the gradual degradation of cognitive and motor functions [19,20]. In contrast, cancer disrupts the delicate balance of cell division, apoptosis, and survival, resulting in the uncontrolled growth of aberrant cells [21,22]. It is speculated that a variety of factors and processes, such as oxidative stress, mitochondrial dysfunction, epigenetic modification, inflammation, metabolic dysregulation, and abnormal cell cycle regulation, play a combined role with varying weights on the basis of the inverse comorbidity between NDs and cancer. This could favor the development of either neurodegeneration or cancer, or both, alternatively or in mutual exclusion [23]. Among pathways and proteins dysregulated in both conditions, the expression of the p53 tumor suppressor is increased in AD and PD [24,25,26] while downregulated in the majority of cancer types [27]. Moreover, the *PIN1* gene is upregulated in several tumors but downregulated in AD [28]. Recently, it has been suggested that this inverse comorbidity may be regulated by non-coding RNAs, activating specific pathways leading to one of the two clinical phenotypes [29]. Hence, exploring the relationship between NDs and cancer could provide new insights into the etiologies and potential therapeutic targets for both conditions.

Herein, this review aims to discuss recent evidence on the role of the main neurodegenerative-associated proteins in cancer development as potential prognostic and diagnostic biomarkers. The challenges and opportunities in targeting these proteins for developing new targeted anticancer drugs will also be explored.

## 2. A Brief Overview of the Main Neurodegenerative Diseases

The term NDs refers to a broad range of heterogeneous neurological disorders affecting the central or peripheral nervous systems and characterized by a progressive and selective process of cellular death affecting neurons [30]. Among them, AD, PD, and ALS are three of the major NDs, and their incidence and prevalence increase rapidly with age, posing substantial issues for public health systems worldwide [31,32]. However, certain disorders can be distinguished by the presence of distinctive symptoms that vary according to the region of the brain where the loss of neurons is occurring [31]. Neuronal deterioration leads to irreversible impairment of cerebral functions, which may manifest as cognitive deficits, dementia, motor alterations, and varied degrees of behavioral and psychological disturbances, depending on the kind of disease [33]. For instance, plaques of amyloid-β (Aβ) and tau tangles are linked to memory loss and cognitive deterioration in AD. PD is characterized by bradykinesia, rigidity, and dopamine-producing neuron loss. ALS causes motor neuron degeneration, which results in gradual muscular weakness and paralysis [34]. With the exception of a small number of familiar cases, the majority of NDs arise from a complex interplay of environmental, genetic, and lifestyle factors [34,35]. Collectively, neuroinflammation, oxidative stress, mitochondrial dysfunction, metabolic dysregulation, and abnormalities in protein quality regulation and degradation are key molecular factors that drive neurodegeneration, leading to chronic cellular stress and ion homeostasis imbalances, resulting in axonal and neuronal death [36,37].

**Figure 1:**
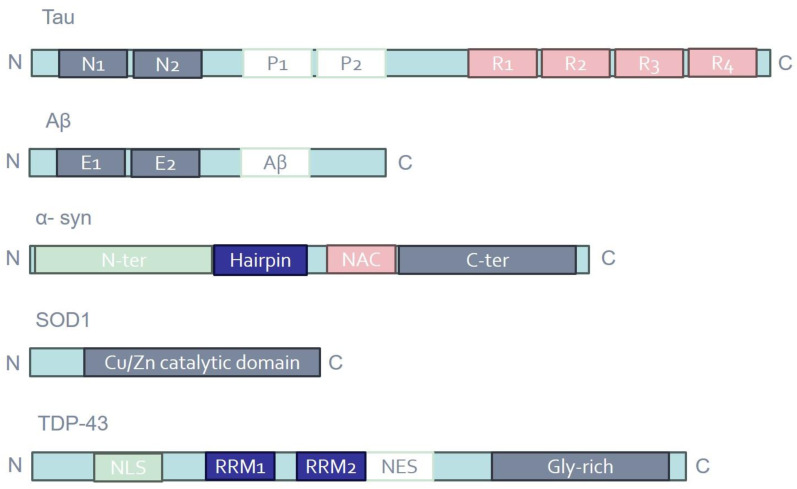
Schematic representation of the domain structures of tau, Aβ, α-syn, SOD1, and TDP-43. Distinct protein domains are indicated in different colors. E1/2; extracellular domains; Gly-rich, glycine-rich region; N1/2, N-terminal regions; NAC, non-amyloid β component; NES, nuclear export signal; NLS, nuclear localization signal; P1/2, proline-rich regions; R1-4, microtubule-binding repeats; RRM1/2, RNA recognition motifs.

Most notably, NDs almost universally share common pathophysiological mechanisms, including protein aggregation (proteinopathy) and the formation of visible inclusion bodies on a neuropathological level [38]. Aggregates and inclusion bodies are observed in surviving neurons within the brain regions undergoing neurodegenerative processes, but their composition varies according to the specific disease [39]. Various proteins are identified based on the neuropathological profile, such as Aβ and tau in AD, alpha-synuclein (a-syn) in PD, and SOD1 and TDP-43 in ALS [40] (Figure 1). However, certain proteins intended to serve as diagnostic biomarkers for a particular ND may also serve as prognostic or diagnostic predictors for other NDs. For instance, elevated levels of total tau (t-tau) and the lowered ratio of phosphorylated tau (p-tau)/t-tau in cerebrospinal fluid (CSF) have emerged as potential diagnostic biomarkers for ALS [41]. Furthermore, the correlation between high CSF t-tau levels and poor survival suggests the possible utility of this biomarker for ALS prognosis [42]. In PD patients, CSF tau levels significantly correlated with cognitive deficits: individuals with high p-tau and p-tau/Aβ42 ratios experienced a deterioration in executive and memory abilities [43]. Additionally, core AD biomarkers can also be used to monitor PD progression. In a longitudinal study, decreased levels of t-tau/Aβ42 and increased p-tau and p-tau/t-tau were found in the CSF of PD patients over a 12-year period [44]. According to Dolatshahi et al., p-tau levels in CSF were low initially but dramatically increased in the PD group during the 1-year follow-up [45]. Similarly, a substantial number of studies reported that CSF total α-syn levels tend to increase in AD patients as compared with controls, appearing to be more indicative of general neurodegeneration than of mechanisms exclusive to AD [46].

## 3. Alzheimer’s Disease (AD)

AD is an ND that progresses over time, predominantly impacting individuals in advanced age, leading to profound deterioration in the quality of life for affected patients [47]. As the disease advances, individuals with AD experience a gradual loss of memory, reasoning, and other cognitive abilities essential for daily functioning [48]. The major pathological hallmarks of AD include brain atrophy, the extracellular deposition of senile plaques composed of insoluble Aβ peptide, and the intracellular formation of neurofibrillary tangles (NFTs) constituted by hyperphosphorylated twisted filaments of the microtubule-associated protein tau in the hippocampus [49,50,51]. Additionally, the presence of these abnormal protein aggregates triggers inflammatory responses and oxidative stress, further exacerbating neuronal damage and contributing to disease progression [52]. Overall, AD represents a significant public health challenge with profound implications for affected individuals and their caregivers.

### 3.1. Tau

Tau is a microtubule-binding protein mainly expressed in neuronal tissue, where it plays a crucial role in cytoskeleton stabilization [53]. Beyond its role in regulating microtubules, tau also participates in other important signaling pathways, including those that control cell proliferation and differentiation, morphogenesis, and motility [54]. In the human central nervous system, six tau isoforms are produced by alternative mRNA splicing of exons 2, 3, and 10 from the microtubule-associated protein tau (*MAPT*) gene [55]. Among three other repeat domains (R1, R3, and R4), exon 10 encodes the second imperfect-repeat microtubule-binding domain (R2) [56]. Depending on the exclusion or inclusion of exon 10, tau proteins become either 3R or 4R tau, respectively, with an approximately 1:1 ratio in the healthy adult human brain [57]. This balance is tightly regulated developmentally, and dysregulation of 3R:4R tau isoforms is associated with the pathogenesis of several NDs [58]. Moreover, tau undergoes a variety of post-translational modifications (PTMs), such as phosphorylation, truncation, methylation, acetylation, glycosylation, nitration, and SUMOylation [59]. Among these PTMs, phosphorylation is the most common, and hyperphosphorylated tau molecules at certain sites reduce the affinity of this protein to microtubules, resulting in neuronal cytoskeleton destabilization and NFT formation [60].

Tau, initially recognized for its significant involvement in neurodegenerative conditions, has recently garnered attention as a crucial contributor to the development of cancer [61]. Aberrantly high expression of tau has been found in various types of tumors, spanning from brain malignancies such as gliomas [54,61] to solid tumors like breast [62,63,64], ovarian [65], gastric [66,67], and prostate cancer [68,69]. The abnormal expression of tau in these tumors is not necessarily linked to the progression of the pathology. For instance, elevated tau expression in a number of carcinoma types, including gliomas, breast cancer, and prostate cancer, has been associated with better patient outcomes [66,70,71], while it appears to be related to a lower prognosis in other cancer forms, like ovarian cancer [72,73].

Surprisingly, tau protein is not just exclusive to neurons; it has also been found in glial cells. Its expression in gliomas, particularly aggressive and incurable types of glial-derived brain cancer, seems to be associated with a better prognosis [61], suggesting that this protein could potentially serve as a valuable prognostic marker in cancer diagnosis and treatment planning [74]. Given its crucial role in the regulation of microtubule stability and dynamics, tau affects the behavior of tumor cells, altering their ability to proliferate and spread throughout the body [74]. Moreover, this protein appears to participate in signaling pathways that regulate the cell cycle and promote survival [75]. By modulating these pathways, tau may induce cancer cell proliferation, contributing to tumor progression and metastasis [76]. Among gliomas, glioblastoma (GBM) is the most aggressive primary brain tumor in the elderly and remains incurable, with a median survival of about 15 months. Interestingly, PTMs in GBM have been studied, suggesting that their regulation may represent a novel therapeutic approach to GBM. Apart from tau hyperphosphorylation inhibitors, other tau-regulating proteins have been identified and are currently undergoing trials for GBM in order to investigate their beneficial effects on therapy [54]. These include the PP2A protein, which promotes tau dephosphorylation; the HAT/HDAC proteins, which can acetylate or deacetylate tau; the OGT/OGA proteins, which transfer or remove GlcNAc from tau; and the HSP70 chaperone system protein, which mediates ubiquitinylation of abnormal tau species for specific degradation [54,77,78,79,80].

Patients who test negative for tau in breast cancer represent just over half of the population, accounting for approximately 49–50% [74,81]. Over the years, multiple studies have established a significant correlation between high tau expression levels and the presence of estrogen (ER)/progesterone (PR) receptors, which play a crucial role in breast cancer pathology. Tumors that exhibit positivity for both ER and tau proteins tend to respond well to hormonal therapy owing to an anomalous estrogen response element [62,82]. Furthermore, studies have shown that the administration of ER inhibitors affects tau expression primarily in cells with elevated tau levels [83,84]. Despite the fact that tau is more common in metastatic breast tumors than in other cancer types, emerging evidence suggests its critical role in enabling tumor cell reattachment through the formation of microtentacles (McTNs), which are dynamic microtubule-based extensions of the plasma membrane [85]. Significant changes in McTN frequency, morphology, and halted cell reattachment were observed with the 3R tau isoform, but future research will examine the impact of specific splice or phosphorylation variants on McTN formation [85]. Notably, breast and ovarian cancer patients receiving paclitaxel display higher tau protein levels than untreated individuals. Therefore, researchers employ elevated tau concentrations as a diagnostic marker to identify hormone-sensitive tumors that are resistant to chemotherapy [86,87,88,89].

Tau has also been found to be overexpressed in gastrointestinal stromal tumors, specifically in Auerbach’s plexus of the small intestine [66,90]. Moreover, short DNA sequences containing an unusually high abundance of CpG dinucleotides, known as CpG islands, have been recognized as crucial functional elements within the genome, governing promoter regions in vertebrates. The progression of colorectal cancer has been linked to the CpG island methylator phenotype (CIMP). In stage II colorectal cancer patients, hypermethylation of tau promoter CpG islands indicates an unfavorable prognosis and serves as a pivotal diagnostic marker for disease progression [91]. Additionally, increased phosphorylation of tau protein, which disrupts microtubule stabilization, has been documented in the SW480 and HCT 116 colorectal cancer cell lines [92]. Enhanced tau expression and phosphorylation, similar to what occurs in some NDs, are associated with the progression and drug resistance reported in various cancers [92,93].

Prostate cancer is the second most frequent cancer in males worldwide, resulting in significant morbidity and mortality. It typically progresses from normal prostate tissue to prostatic intraepithelial neoplasia before advancing to malignancy [94]. While surgical resection and radiotherapy are common treatments, metastasis often occurs upon disease recurrence. Androgen deficiency therapy is frequently employed for metastatic cases due to the crucial role of androgen signaling in tumor growth, given the expression of androgen receptors in prostate tumors [95]. Some authors found that cancerous prostate cells express hyperphosphorylated tau and multiple protein isoforms [68]. In another study, it was demonstrated that the phosphorylated tau form at residue Thr231 is restricted to the G2/M cell cycle phase [69]. According to this finding, the phosphorylation status of tau protein is a crucial marker of the G2/M phase in prostate cancer cells, and altering tau phosphorylation may impair the ability of the cell to proceed through the phase more quickly [69].

The association between tau and chemoresistance, particularly to drugs like taxanes, adds another layer of complexity. Taxanes, which are extensively used in the treatment of various tumor types such as breast, ovarian, and gastric carcinomas, exert their effects by targeting microtubules [65,93]. However, the presence of tau, which shares the same binding site on microtubules as taxanes, can confer resistance to these drugs, making treatment more challenging [96]. Overall, the emerging understanding of the multifaceted roles of tau in cancer highlights its importance as a possible target for therapeutic intervention. Further research into the precise mechanisms underpinning the involvement of tau in tumorigenesis holds promise for developing novel strategies to treat cancer and improve patient outcomes.

### 3.2. Aβ

As mentioned above, the Aβ protein has been associated with the formation of senile plaques in the brain, a hallmark of AD pathology [49,97]. However, the exact role of Aβ and its mechanisms of action are not yet fully understood. The Aβ derives from the sequential cleavage of the amyloid precursor protein (APP) and can be a peptide of 40 (Aβ40) or 42 (Aβ42) amino acids [98,99]. Besides differences in size, Aβ can undergo several PTMs, such as phosphorylation, oxidation, glycosylation, nitration, racemization, isomerization, and pyroglutamylation, resulting in a variety of peptides with different physiological or pathological properties [100]. Similarly, APP is also subjected to a variety of PTMs that regulate its location and trafficking throughout the cell, including phosphorylation, proteolytic processing, glycosylation, and sulfation [101].

Compared to tau protein, the exact role of Aβ in cancer is still being investigated and debated by scientists. The majority of studies have shown an elevated expression of APP in several types of tumors, including breast, pancreatic, prostate, and colon cancer, promoting cellular growth and proliferation [102,103,104,105]. Additionally, high levels of Aβ have been observed in patients affected by primary brain tumors [106,107,108]. Conversely, it has been demonstrated that non-toxic oligomers of Aβ can promote the death of tumor cells in human hematological (NB4) and solid (A549, lung, and MCF-7, breast) cancer cell lines, suggesting its potential protective role against tumors. In particular, the authors demonstrated that peptides of Aβ, particularly those with a high antiparallel β-sheet organization but a relatively low amount of β-sheet structures, are able to inhibit the growth of hematological and solid cancer cells, highlighting the importance of the structural features of amyloid species [109].

In breast cancer, it has been discovered that APP promotes tumor growth and metastasis [110,111,112]. Indeed, its overexpression is associated with increased invasiveness and the recurrence of breast cancer [113]. According to studies, APP interacts with a variety of signaling pathways, including the mitogen-activated protein kinase (MAPK) pathway, which contributes to cancer progression [110,114]. Moreover, APP expression is influenced by androgen receptor activity and is associated with a poor prognosis in some breast cancer subtypes [115]. Inhibiting APP expression or its processing products, such as soluble amyloid precursor protein alpha (sAPPα), can reduce breast cancer cell migration and proliferation. These findings highlight the potential advantages of targeting APP-related pathways in breast cancer therapy [116].

In glioma and GBM cell lines, inflammatory markers like cyclooxygenase-2 (COX-2), cytosolic phospholipase, interleukin-1β (IL-1β), and APP have been found to be overexpressed, suggesting a possible link between inflammation and disease progression [117]. Additionally, GBM has been associated with increased mortality in individuals with AD [6,118,119]. Immunostaining studies in animal models found Aβ42 deposits in glioma tumors and blood vessels. The use of thioflavin has improved the detection of aggregated Aβ in glioma tumors [107]. Furthermore, amyloid precursor-like protein 2 (APLP2) has emerged as a relevant factor in GBM, with its involvement documented in a variety of cellular processes and its association observed with metastasis, cell proliferation, and invasion across multiple cancer types, including breast, pancreatic, lung, and colon cancer [120,121]. APLP2 was also reported to be highly expressed in pancreatic cancer cell lines, especially the APLP2 C-terminal fragment and the APLP2-modified glycosaminoglycan in relation to APP full-length and C-terminal fragments [122]. APLP2 is able to alter the actin cytoskeleton and promote pancreatic cancer growth and metastasis [121]. Studies have found a relationship between APP and androgen-responsive genes in prostate cancer [103], implicating APP in malignancy [123]. Research has shown that APP affects prostate cancer cell proliferation and migration, with higher APP levels associated with increased migratory activity and the expression of genes linked to metastasis [123].

Regarding other types of tumors, studies have been conducted on colorectal cancer, nasopharyngeal carcinoma (NPC), hepatocellular carcinoma (HCC), and non-small cell lung carcinoma (NSCLC) [113]. In colon cancer, carbamazepine (CBZ), a drug with antiepileptic properties, and valproic acid (VPA) have emerged as promising therapeutic options. Recent studies indicate that both CBZ and VPA exhibit anticancer effects by reducing APP levels in human colon cancer cells [113,124]. Concerning NPC, the expression of APP is elevated in NPC tissues, and patients treated with radiotherapy exhibit higher APP levels, raising the possibility that APP could serve as a helpful biomarker for prognosis and diagnosis in NPC patients [22]. Additionally, APP contributes to the invasion and migration of NPC cells. An in vitro study demonstrated that APP silencing significantly inhibits the epithelial–mesenchymal transition (EMT) of NPC cells through the downregulation of the MAPK signaling pathway [125]. Interestingly, the regulation of APP is mediated by histone deacetylase in HCC, suggesting the importance of epigenetic modifications in the development of cancer [126]. Finally, it has been found that APP and, in particular, its phosphorylated form are potent prognostic factors in NSCLC [127].

## 4. Parkinson’s Disease (PD)

PD is a chronic progressive ND that primarily affects the nervous system, leading to motor and non-motor symptoms that significantly impair patients’ quality of life [128,129,130]. The etiology of PD is complex and multifactorial, involving a combination of genetic, environmental, and neurochemical factors [129]. One of the key hallmarks of PD is the progressive degeneration of dopaminergic nerve cells in the substantia nigra of the brain, which results in dopamine loss in the striatum, a brain region involved in the control of voluntary movement [131,132]. Despite advances in understanding the pathogenic mechanisms of PD and in the development of symptomatic treatments to alleviate motor symptoms, the long-term management of the disease remains challenging, with limited therapeutic options available to slow or halt disease progression.

**Figure 2:**
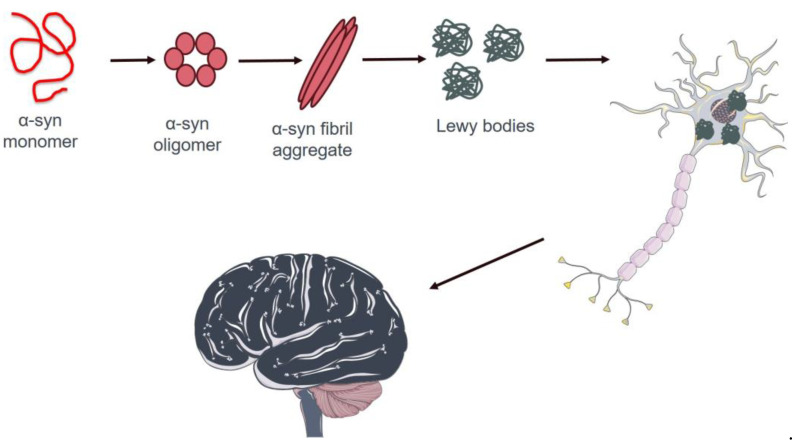
Schematic representation of pathological protein aggregation of α-syn in neuron cells in the brain of patients affected by PD. Proper α-syn formation is essential for neuronal function and tissue health. In patients with PD, this protein aggregates into protein fibers known as Lewy bodies. These aberrant structures occur in the cytoplasm of neurons, causing a pathological condition that leads to severe nervous system degeneration and, ultimately, cell death. The image was created using Servier Medical Art modified templates, licensed under a Creative Commons Attribution 3.0 Unported License (https://smart.servier.com, accessed on 10 April 2024).

Alpha-syn has garnered considerable interest in PD research due to its central role in disease pathogenesis [133]. It is a protein rich in alpha-helical repeat sequences that are widely expressed in the nervous system, particularly in presynaptic neurons. Although the exact physiological function of α-syn is not fully understood, it is believed to play a role in regulating synaptic vesicle release and reuptake. However, it has become increasingly evident that α-syn is also involved in the formation of insoluble protein aggregates, known as Lewy bodies, which are a hallmark clinical feature of PD [134] (Figure 2). These protein aggregates accumulate inside nerve cells, impairing their function and causing progressive deterioration of the nervous system [135]. Numerous studies have investigated the mechanisms underlying the formation of α-syn aggregates and their role in causing neuronal death in PD [136]. A deeper understanding of these processes may lead to new therapeutic strategies aimed at preventing or slowing the progression of PD [137]. The aggregation and stability of α-syn may be influenced by several PTMs, including phosphorylation, ubiquitination, truncation, acetylation, SUMOylation nitration, *O*-GlcNAcylation, and C-terminal cleavage [138].

### 4.1. Alpha-Syn

In recent years, new research avenues have been pursued concerning this protein, which is typically studied in the neurological field, particularly with several studies investigating its relevance in oncological diseases. The syn family has been shown to be involved in oncogenic pathways, which accelerate cellular processes leading to cancer onset. A study demonstrated that α-syn has a role in the development of pancreatic ductal adenocarcinoma (PDAC). Researchers found that α-syn is overexpressed in 20 cases of cancer patients with two types of this tumor. However, this was more prominent in PDAC samples with perineural infiltration than in tumors without perineural infiltration [139].

The involvement of α-syn in the oncological field is not limited solely to the aforementioned tumor. Melanoma has also been found to express this protein [140,141]. In particular, researchers discovered overexpression of this abnormal protein through numerous preclinical studies. Shekoohi and colleagues demonstrated that inhibiting α-syn expression reduces the development of melanoma tumor cells [141]. This evidence suggests that α-syn may have an active role in oncogenesis rather than just being an anomalous biological phenomenon [141]. Another potential role of this marker has been elucidated: the loss of α-syn expression in skin cancer cells altered iron metabolism regulation, resulting in cells with reduced TfR1 and FPN1 and increased ferritin and DMT1 levels [141]. Overexpression of α-syn in melanomas is thought to be associated with significant alterations in autophagy. However, further investigations will be needed in this regard [142]. This protein is highly expressed in both primary and metastatic melanomas, indicating its association with these types of skin cancer. On the other hand, it is notably absent in non-melanocytic cutaneous carcinoma and healthy skin samples, suggesting a specific role for α-syn in melanoma development and progression [143]. Importantly, the Ser129 phosphorylated form of α-syn plays a role in the transition of this protein to pathogenic species not only in PD but also in melanoma, where it localizes in the extracellular space of melanoma cells [143,144].

The increase in α-syn levels plays a significant role in promoting the aggressive characteristics of meningiomas by activating the Akt/mTOR pathway. This finding suggests that targeting α-syn could offer a promising therapeutic strategy for treating malignant meningiomas [145].

### 4.2. Other PD-Related Proteins

In addition to α-syn, other PD-associated proteins are also involved in some types of cancer. Among them, ubiquitin carboxyl-terminal hydrolase L1 (UCHL1), also called PARK5, is expressed widely throughout the brain and plays an important role in the ubiquitin–proteasome system (UPS), functioning as a hydrolase or de-ubiquitinase enzyme [146]. Although this protein is normally expressed primarily in neurons, it not only causes NDs but also displays a complex involvement in the development and progression of cancer by acting as a tumor suppressor or oncogenic factor depending on different cancer types [147,148]. *UCHL1* undergoes promoter methylation, which in turn causes the progression of many malignancies, including HCC, NPC, head and neck squamous cell, gastric, ovarian, breast, and pancreatic neuroendocrine cancers [147]. When *UCHL1* expression is restored, it controls important cyclin levels (like p53), prevents cancer cells from proliferating, and promotes their apoptosis [149,150]. On the other hand, UCHL1 acts as an oncogenic factor via PI3K/Akt, MAPK/Erk, and other signaling pathways to promote the development, invasion, and metastasis of breast cancer as well as NSCLC, lymphoma, osteosarcoma, and neuroblastoma [147].

Other proteins that are involved in PD pathogenesis are DNAJ/HSP40 molecular chaperones, as evinced by genetic studies in rare familial forms of PD [151]. Through the J domain, DNAJ proteins bind to Hsp70 and promote its ATPase activity [152]. Among members of the DNAJ family, a whole transcriptomic analysis revealed an upregulation of *DNAJC14* in osteosarcoma, suggesting its potential role also in cancer [148].

## 5. Amyotrophic Lateral Sclerosis (ALS)

ALS, also known as Lou Gehrig’s disease, is a progressive ND that primarily affects the motor neurons in the brain and spinal cord. This devastating condition leads to the gradual degeneration and death of these motor neurons, causing a progressive loss of voluntary muscle control [153,154]. As a result, individuals with ALS experience muscle weakness, twitching, and eventually paralysis. To date, more than 50 modified genes have been examined in ALS, but they are primarily linked to mutations in the *SOD1*, *TARDBP*, and *FUS*/*TLS* genes, encoding Cu/Zn superoxide dismutase-1, transactive response DNA-binding protein 43 (TDP-43), fused in sarcoma/translocated in liposarcoma, respectively [39,153]. These proteins are involved in a broad range of cellular pathways, including mitochondrial dysfunction, excitotoxicity, autophagy with protein homeostasis loss, inflammation, DNA damage repair, aberrant RNA metabolism, and impaired intracellular trafficking [155].

### 5.1. SOD1

**Figure 3:**
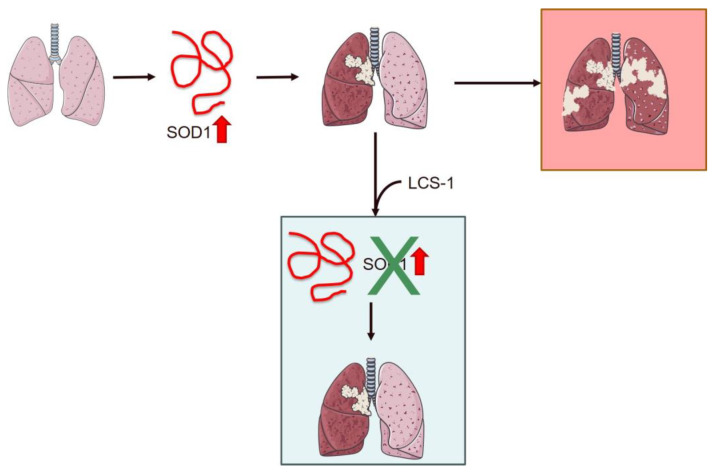
Schematic representation of the role of SOD1 overexpression in lung cancer and the effect of treatment with the inhibitor LCS-1 on cancer progression. Overexpression of SOD1 promotes metastasis in NSCLC patients. Treatment with LCS-1 blocks SOD1 expression, decreasing tumor growth. The red arrow indicates SOD1 overexpression. The image was created using Servier Medical Art modified templates, licensed under a Creative Commons Attribution 3.0 Unported License (https://smart.servier.com, accessed on 10 April 2024).

In 1993, research identified ALS-associated mutations in the *SOD1* gene located on chromosome 21. This gene encodes the Cu/Zn superoxide dismutase protein, a vital enzyme within the cytoplasm responsible for neutralizing superoxide radicals into hydrogen peroxide and molecular oxygen [39,156,157]. SOD1 is ubiquitously expressed, highly conserved, and accounts for approximately 1% of all proteins in the cytoplasm of cells [158]. Protein function and stability are regulated by several PTMs on key amino acid residue side chains, including phosphorylation, lysine modifications, redox modifications, and nitration [159]. Regarding the involvement of this protein in oncological pathogenesis, recent studies have shown that SOD1 is altered and overexpressed in tumors, and it appears to be able to promote the metastasis of tumor masses. Many studies have been published in the search for inhibitors in lung cancer, in particular NSCLC [160,161,162]. Somwar et al. made a significant discovery when investigating inhibitors that target molecules commonly found in tumor cells. Among them, LCS-1 was distinguished for its ability to inhibit SOD1, successfully blocking apoptosis while promoting cell growth. This finding underscored LCS-1 as a potential therapeutic candidate, particularly in counteracting tumor progression [160] (Figure 3). Furthermore, their research demonstrated that the administration of CSF-1 could effectively suppress the overexpression of SOD1, slowing tumor growth in lung cancer. Notably, their study extended beyond lung cancer to highlight LCS efficacy in combating breast cancer [160].

Intriguingly, the role of SOD1 in oncological biology is not limited to lung cancer alone. Indeed, breast tumors also exhibit high levels of SOD1, encouraging studies into pharmacological interventions aimed at reducing its overexpression [160,163,164]. Even with this type of tumor, research has focused on the study of pharmacological molecules aimed at inhibiting the overexpression of SOD1 in breast cancer cells. In-depth investigations of erbB2 (MMTV-iErbB2) and Wnt (MMTV-Wnt) inducible transgenic breast cancer mouse models revealed a strong correlation. Inhibiting SOD1 resulted in a significant reduction in tumor proliferation, particularly evident in samples treated with LCS-1. These promising findings were further confirmed in cell line studies, highlighting the therapeutic potential of targeting SOD1 in breast cancer management [165]. The mentioned research confirms that SOD1 plays a role in tumor cell proliferation and hence represents a promising target for anti-tumor chemical compounds. The discovery that SOD1 plays a crucial role in the initiation of tumors correlates with a subsequent report that mTORC1 controls SOD1 via phosphorylation, suggesting that increased SOD1 under starvation conditions is required for enhancing cancer cell survival and contributing to chemoresistant mechanisms [166].

Along with LCS-1, clinical and preclinical studies are also investigating other inhibitory compounds, such as ATN-224, a copper chelator compound that has been examined in the field of oncology. In particular, there are clinical trials involving patients with prostate cancer. However, no clear and definitive results have yet been obtained in these studies, so further research is required to fully understand the role of SOD1 in oncological diseases. Additionally, future studies on this protein will be essential for developing new drugs useful for cancer research [161,167,168,169].

### 5.2. TDP-43

TDP-43 was first identified in 1995 when it was found to bind the transactivation response region (TAR) of HIV DNA, hence the name TAR DNA binding protein. Soon later, it was discovered in the human brain and various cell culture systems [39,170]. TDP-43 has proven to be a protein of significant interest due to its highly conserved nature across species and widespread presence in both human and rodent cells, where it is predominantly found in the nucleus. TDP-43, consisting of 414 amino acids and weighing 43 kDa, is the protein product of the *TARDBP* gene located on chromosome 1. It belongs to the heterogeneous ribonucleoprotein (hnRNP) family, which is a varied group of RNA-binding proteins [171,172,173]. Despite its ubiquitous presence, the exact cellular function of TDP-43 is unknown, although numerous studies have revealed its involvement in biological processes, including the regulation of gene transcription, modulation of splicing events, and preservation of mRNA stability [174]. Aberrant aggregation and mislocalization of TDP-43 are associated with different classes of PTMs that can influence its functionality and cellular status. Among them, phosphorylation, formation of C-terminal fragments, disulfide bridge formation, acetylation, ubiquitination, and SUMOylation are extensively investigated [175].

Delving into some research conducted studying TDP-43 in certain types of tumors, Lin and colleagues focused their study on GBM. In particular, it was found first that exogenous overexpression of TDP-43 activated autophagy and prevented stress-induced apoptosis, and that histone deacetylase 6 (HDAC6) was involved in the activation of autophagy in these cells. Following these results, an HDAC6 inhibitor, SAHA, was used, which demonstrated a significant reduction in the phenomenon activated by TDP-43 and HDAC6. Also, in this intriguing study, it emerged from experiments performed on patient biopsies that TDP-43 and HDAC6 were negatively correlated and colocalized in tumor lesion sites [176].

In addition to GBM, TDP-43 is also implicated in HCC, although its role is not yet fully understood. In one study, it was shown that TDP-43 is overexpressed in both clinical samples of patients with this condition and in HCC cell lines, resulting in changes in cell proliferation and metastasis formation. XAV939, an inhibitor of the Wnt/β-catenin signaling pathway, effectively blocked TDP-43 overexpression. This study revealed how TDP-43 may act and the signaling pathways it uses, which makes it a possible future treatment target [177].

Recently, the importance of studying TDP-43 has also emerged in skin tumors, particularly melanoma. A study revealed that TDP-43 is overexpressed in samples from patients with melanoma. This significant increase in TDP-43 has been found to be correlated with patient mortality. This study demonstrated that silencing this protein significantly inhibited cell proliferation and metastasis in the A375 and WM451 cell lines. The results confirm prior studies on different tumors, suggesting that blocking the overexpression of TDP-43 lowers cell proliferation and metastasis formation [178].

Another notable example of the involvement of this important neurological biomarker in tumors is breast cancer research. In this context, aberrant alternative splicing is recognized as a hallmark of malignancy. In 2018, TDP-43 was identified as a splicing regulator responsible for triple-negative breast cancer (TNBC). Researchers discovered an overexpression of TDP-43 in this tumor type, which leads to a poorer prognosis for TNBC. This study also demonstrated how silencing TDP43 can halt tumor progression and metastasis, whereas overexpression of TDP43 has the opposite effect, enhancing the malignancy of mammary epithelial cells [179]. Furthermore, it has been discovered that the role of TDP-43 in splicing also involves CD44. This is a hallmark of breast cancer stem cells (BCSCs), and the influence of their alternative splicing is believed to play an important role in the rapid progression of the oncological pathology [179,180]. Kim and colleagues also found that in some cases of breast cancer, the overexpression of TDP-43 is important for the apoptosis induced by TRIM16, a member of the tripartite motif protein family that acts as a potential tumor suppressor [181].

However, despite the very interesting results about the involvement of TDP-43 in tumors, there is a significant need for future research to fully understand how this typically neurological marker works in cancer.

## 6. Other Neurological Biomarkers in Cancer Disease

In this chapter, we will discuss additional biomarkers associated with neurological disorders that are not conventional NDs. However, some other conditions show markers that have emerged to play a significant role in the field of tumors and have been the topic of recent oncological research.

### 6.1. Double Homeobox 4 (DUX4)

DUX4 is the biomarker typically associated with a form of muscular dystrophy, namely facioscapulohumeral muscular dystrophy (FSHD). DUX4 is expressed in the early stages of development in stem cells and germ cell lines, while it is repressed through a methylation mechanism mediated by repeats [182,183] occurring during cellular differentiation [184] and in most somatic tissues, including muscles [182,185,186], except for the thymus [187] and keratinocytes [188]. DUX4 toxicity is related to different PTMs, such as phosphorylation, methylation, and acetylation [189].

Its overexpression has historically been linked to muscular dystrophy and other muscle changes [182,190]. However, in recent years, a number of studies have linked this marker to oncological pathologies. In 2019, a study showed alterations in blood tumor samples [191]. This was confirmed by several other reports, which found that altered DUX4 with genetic mutations could serve as a marker for the development of acute lymphoblastic leukemia [192,193,194]. It has also been demonstrated that this marker is altered in round-cell sarcoma or Ewing-like sarcomas [195]. However, in an aggressive type of sarcoma that primarily affects pediatric and young patients, the causal event is a fusion between the high mobility group (HMG) box, which contains the capicua protein (CIC), and DUX4. In a non-pathological state, CIC acts as a transcription repressor, but when fused with DUX4, it becomes a transcription activator [196,197,198]. CIC-DUX4 induces small round-cell sarcomas distinct from Ewing sarcoma. All this has led to the understanding that CIC-DUX4 can become an oncogene. However, altered DUX4 also appears to function as a tumor suppressor in synovial sarcoma and colon cancer [199,200]. However, the role of altered DUX4 expression in various types of tumors requires further specific and in-depth studies, as all studies so far are relatively recent. In the future, new evidence may emerge on how this marker acts in cancer, and these findings may also be useful in neurobiological research for the development of definitive therapies for FSHD.

### 6.2. Neurofilament Light Chain (NfL)

The neurofilament light chain (NfL) is an essential structural component of neurons belonging to the neurofilament family, along with the neurofilament medium chain (NfM) and the neurofilament heavy chain (NfH). These proteins are crucial for maintaining the structural integrity of axons and facilitating intracellular transport within the nervous system [201]. NfL undergoes different PTMs, including phosphorylation, O-linked glycosylation, nitration, and ubiquitination [202]. In recent years, NfL has garnered increasing attention as a potential biomarker for a wide range of neurological diseases, including AD, PD, and ALS [203]. Its relevance stems from the fact that, following neuronal injury or degeneration, NfL is released into CSF and blood, allowing its measurement as an indicator of neuronal damage [204,205]. NfL has been extensively studied in the context of neurological diseases, in particular, multiple sclerosis (MS). In patients with these conditions, elevated levels of NfL in CSF and blood have been correlated with disease severity, symptom progression, and neurological deterioration [205]. In MS, elevated levels of NfL have been associated with increased disease activity and greater brain volume loss, making it a promising biomarker for monitoring treatment response and disease progression over time [205]. Despite the potential of NfL as a biomarker, there are some challenges and limitations to consider. For example, NfL levels can vary significantly between healthy individuals and patients with neurological diseases, making it difficult to establish a universal diagnostic threshold [206,207]. Additionally, factors such as age, sex, and the presence of comorbidities can influence NfL levels and complicate the interpretation of the results. NfL continues to be the focus of intense research for its potential in early diagnosis, monitoring disease progression, and assessing treatment response in neurological diseases. Further studies are needed to refine measurement methods, better understand individual variations, and identify complementary biomarkers that can improve the specificity and accuracy of disease diagnosis and monitoring [208]. While historically studied in the context of neurological diseases, recent research has shed light on the potential involvement of NfL in cancer [209]. NfL has been identified in various types of solid tumors, suggesting a broader role beyond its traditional neurological context [210,211,212]. Efforts are focused on assessing NfL expression in tumors and its correlation with clinical and prognostic parameters [209,213]. Additionally, research is focused on identifying the molecular mechanisms underlying the regulation of NfL expression in tumors and developing new targeted therapeutic strategies that target NfL and its pro-tumorigenic effects.

Recently, a study examined the expression levels of this marker in the blood of NSCLC patients. Specifically, researchers evaluated NfL in serum from subjects with and without brain metastases to determine whether its levels were associated with metastasis development. The most interesting finding of this study was the presence of a high level of NfL in the serum prior to the patient’s diagnosis of brain metastases. Furthermore, it emerged that the level of NfL at the time of diagnosis of brain metastases was correlated with survival [214]. These results are consistent with previous studies [204,215].

NfL was also tested as a biomarker for axonal damage severity in chemotherapy-induced peripheral neuropathies. Peripheral neuropathy is a common complication of chemotherapy, with symptoms ranging from minor to severe, potentially affecting patients’ quality of life and ability to finish treatment cycles. In this context, identifying reliable biomarkers for peripheral neuropathy could be crucial for better predicting and managing this complication. Research has progressed with several studies investigating NfL levels in the blood of patients with gynecological tumors treated with paclitaxel, a routinely used chemotherapeutic drug for this type of tumor [216]. This study revealed an association between serum NfL levels and the severity of paclitaxel-induced peripheral neuropathy in patients with gynecological tumors. Higher levels of serum NfL were observed in patients who developed more severe peripheral neuropathy while receiving paclitaxel. These findings suggest that serum NfL levels could be used as a biomarker to assess the risk and severity of peripheral neuropathy in patients with gynecological tumors undergoing chemotherapy [211,212,216,217].

## 7. Blood-Based Biomarkers for Early Diagnosis and Surveillance of Cancer

Early diagnosis and effective monitoring of cancer have long been a priority in the field of oncology [218,219]. Traditional diagnostic methods, such as imaging and tissue biopsies, while essential, have significant limitations: they often detect cancer only after it has reached a specific size or stage, potentially delaying diagnosis [220]. Additionally, these procedures can be costly, require specialized equipment, and may not always be accessible in resource-limited settings. Tissue biopsies, while providing definitive pathological confirmation, are invasive, might cause discomfort or complications, and may not always be feasible depending on the tumor’s location or the patient’s health status [221]. In recent years, advancements in biomarker research have highlighted the potential of blood-based biomarkers as an essential resource for both early cancer detection and on-going surveillance [222]. These biomarkers offer a less invasive, more cost-effective, and potentially earlier detection method compared to traditional techniques. Blood-based biomarkers are measurable indicators found in the blood that may suggest the existence of cancer. They include proteins, nucleic acids, exosomes, and metabolites. The measurement of these biomarkers in blood samples provides a non-invasive, easily accessible approach to monitoring disease occurrence and progression, potentially offering real-time insights into the biological behavior of malignancies [223,224].

As previously stated, Aβ, tau, α-syn, SOD1, and TDP-43 are key biomarkers in NDs and have been associated with cancer. Aβ peptides can influence cellular processes such as apoptosis and inflammation [109]. Elevated levels of Aβ in the blood have been observed in some cancer patients, suggesting that this protein may serve as a biomarker for cancer diagnosis and monitoring [225]. Tau is also expressed in various malignancies, where it may impact cell structure and division. Blood-based assays for tau could thus be a useful tool for early cancer detection as well as evaluating disease progression and therapy response [61]. Alpha-syn is also found in several cancer types, where it may influence cell proliferation, metastasis, and chemoresistance [226]. The detection of α-syn in blood samples could be utilized for early cancer detection and to monitor the effectiveness of therapeutic interventions [226,227]. Concerning SOD1, measuring its levels in the blood could provide insights into the oxidative stress status of cancer cells, making this protein a possible biomarker for cancer diagnosis and monitoring [228]. Altered SOD1 expression has also been observed in various cancers, possibly contributing to tumor growth and resistance to therapy [229]. As already mentioned, studies have identified abnormal TDP-43 expression in various cancers, suggesting it may play a role in tumorigenesis. TDP-43 has the ability to bind RNA and regulate gene expression, implicating it in cellular processes such as proliferation and apoptosis [230]. The presence of TDP-43 in the blood of cancer patients could serve as a biomarker for early cancer detection and monitoring disease progression.

Future research should focus on integrating these biomarkers into clinical practice and combining them with other diagnostic tools to create a comprehensive, multi-modal approach to cancer diagnosis and surveillance. The development of standardized protocols for biomarker detection and quantification will be essential for their widespread implementation in oncology.

## 8. Conclusions and Future Directions

The growing evidence of the involvement of typical biomarkers of NDs in the oncological context (summarized in Table 1) opens new avenues in research and clinical practice. These proteins also play a role in many tumor-related signaling pathways leading to cell cycle progression, proliferation, invasion, epithelial–mesenchymal transition (EMT) activation, tumor growth, metastasis formation, and chemoresistance. From a translational perspective, these results offer several pertinent implications for preventive and treatment strategies for both conditions. For instance, the risk of the development of NDs and cancer will be reduced with strategies that slow aging. A healthy lifestyle and metabolism have been demonstrated to reduce the risk of cognitive impairment and cancer [231,232,233]. Related therapies are also known to decrease oxidative stress, enhance mitochondrial function, and lower inflammatory marker levels [234]. Moreover, medications that target different signs of aging are also being investigated in both fields. Metformin, a biguanide used to treat type 2 diabetes, was found to reduce the risk of dementia, cancer, and other age-related disorders [235,236] by reducing insulin and IGF-1 levels, inhibiting the mTOR pathway, blocking mitochondrial function, reducing oxidative damage, and activating the AMP kinase [237,238,239].

Different therapeutic strategies targeting ND biomarkers may also be used for cancer. These approaches can consist of targeting these proteins indirectly, through the regulation of post-translational modifications, or directly by inhibiting their aggregation, using active and passive immunotherapies, reducing their cell levels with antisense oligonucleotides, or inducing their clearance via the autophagy–lysosomal system [54,240].

However, it is important to note that the specific role of these biomarkers in tumors and their mechanisms of action remain subjects of ongoing study. Some connections between neurodegeneration and cancer may suggest that they should co-occur, while others could help explain the pattern of inverse comorbidity shown in epidemiologic research. This contradictory picture could potentially shed light on why certain tumors have an inverse relationship with neurodegeneration while others do not. Further research is needed to fully understand the clinical implications of this interconnection and to develop targeted diagnostic and therapeutic strategies that can harness the potential of these biomarkers in the oncological context. Additionally, considering the heterogeneity of tumors and the importance of larger and longitudinal studies to confirm and further explore present findings is essential. Ultimately, integrating knowledge of NDs into oncological research could provide important insights toward improving the management and treatment of cancer patients.

## Figures and Tables

**Table 1:** A summary of altered ND-related protein in each cancer type.

Altered Proteins (Main Effects)	Associated NDs	Cancer Types (Main Effects)	References
Tau (cellular dysfunction and neuronal cell death)	AD	Gliomas (interaction with cancer-related kinase proteins promoting cell proliferation, migration, and survival)	[54,61]
		Breast cancer (tumor cell reattachment through the formation of McTNs and chemoresistance)	[62,63,64,85]
		Ovarian cancer (cell proliferation and chemoresistance)	[65]
		Gastrointestinal stromal cancer (cell progression, metastases, and chemoresistance)	[90]
		Colorectal cancer (cell migration and invasion)	[91,92]
		Prostate cancer (cell cycle progression and chemoresistance)	[68,69]
APP/APLP2/Aβ (inflammation, synaptic dysfunction, excitotoxicity, and oxidative stress)	AD	Pancreatic cancer (cell proliferation, tumor growth, and metastases)	[102,121,122]
		Breast cancer (cell proliferation, migration, invasion by the MAPK signaling pathway)	[104,110,112,114,115,116]
		Gliomas (tumor growth)	[106,107,117]
		Prostate cancer (cell proliferation)	[103]
		Colon cancer (tumor growth)	[105]
		Nasopharyngeal carcinoma (EMT activation)	[125]
		Hepatocellular carcinoma (cell survival)	[126]
		Non-small cell lung carcinoma (metastases)	[127]
α-syn (neurotoxicity resulting in neuronal cell death)	PD	Pancreatic ductal adenocarcinoma (cell invasion)	[139]
		Melanoma (cell growth)	[140,141,142,143,144]
		Meningioma (cell progression via the Akt/mTOR pathway)	[145]
SOD1 (excitotoxicity, oxidative stress resulting in motor neuron death)	ALS	Non-small cell lung carcinoma (cell growth and metastases)	[160,162]
		Breast cancer (cell proliferation and metastases)	[163,164,165]
TDP-43 (neuronal toxicity and dysfunction)	ALS	Glioblastoma (tumor progression by promoting autophagy)	[176]
		Hepatocellular carcinoma (EMT activation and metastases by Wnt/β-catenin pathway)	[177]
		Melanoma (cell proliferation and metastases by modulation of glucose metabolism)	[178]
		Breast cancer (tumor growth, progression, and stemness)	[179,180,181]
DUX4 (muscle cell apoptosis, inflammation, oxidative stress resulting in muscle wasting)	FSHD	Acute lymphoblastic leukemia (leukemogenesis by deregulation of the ERG oncogene)	[192,193,194]
		Ewing-like sarcomas (cell cycle progression and metastases)	[195,197,198]
		Synovial sarcoma (cell death acting as tumor suppressor)	[199]
		Colon cancer (decrease in cell proliferation upon *NFE2L3* silencing)	[200]
NfL (axonal degeneration)	AD, PD, ALS, MS	Head and neck cancer (methylation-mediated chemoresistance)	[210]
		Ovarian cancer (biomarker of paclitaxel-induced peripheral neuropathy)	[211]
		Breast cancer (biomarker of paclitaxel-induced peripheral neuropathy)	[212,217]
		Non-small cell lung carcinoma (biomarker for brain metastases)	[214]
